# Food-Based Strategies for Depression Management From Iranian Traditional Medicine Resources

**DOI:** 10.5812/ircmj.14151

**Published:** 2014-02-05

**Authors:** Mandana Tavakkoli-Kakhki, Malihe Motavasselian, Mahmoud Mosaddegh, Mohammad Mahdi Esfahani, Mohammad Kamalinejad, Mohsen Nematy

**Affiliations:** 1 Department of Traditional Medicine, Shahid Beheshti University of Medical Sciences, Tehran, IR Iran; 2 Department of Traditional Medicine, Tehran University of Medical Sciences, Tehran, IR Iran; 3 Department of Pharmacognosy, Shahid Beheshti University of Medical Sciences, Tehran, IR Iran; 4 Department of Nutrition, School of Medicine, Biochemistry and Nutrition, Endoscopic and Minimally Invasive Surgery, and Cancer Research Centers, Mashhad University of Medical Sciences, Mashhad, IR Iran

**Keywords:** Medicine Traditional, Depression, Diet

## Abstract

**Background:**

Considering the increasing prevalence of depression in contemporary societies, general tendency for safer treatments with fewer side effects has recently been a subject of interest.

**Objectives:**

Food-based strategies, which are one of the outstanding medical solutions in Complementary and Alternative Medicine including Iranian Traditional Medicine have been investigated.

**Materials and Methods:**

In this review study, firstly some important sources of Iranian Traditional Medicine including Kamel al-Sanaat al-Tibbyyah, Al-Qanun fi al-Tibb and Zakhireh Kharazmshahi were reviewed. Next, a literature search was performed on PubMed and Magiran databases with the keywords “depression”, “depressive”, “mood”, “antidepressant”, “antidepressive”, “nutrition”, “nutritional”, “diet”, “meal”, “food”, “functional food”, “healthy food”, “healthy diet”, “medicinal food” and scientific and English terms of all singular foodstuff and some combined foodstuff which are introduced in this paper.

**Results:**

Food-based strategies for depression management in Iranian Traditional Medicine resources involving both prevention and treatment parts have been classified under three headings singular foodstuffs, combined foodstuffs, and nutrition rules with the separation of prohibition and prescription items. Among the prescribed or the prohibited singular and combined foodstuffs in Iranian Traditional Medicine manuscripts, only the effectiveness of fish, garlic, milk, oregano, mint, and spinach on depression has been examined by modern medicine methods.

**Conclusions:**

The presented food-based strategies in this study introduce a precise management for depression benefiting from Iranian Traditional Medicine Resources.

## 1. Background

Considering the high prevalence of mood disorders in today’s societies (1), some significant complications of depression such as increased risk of cardiovascular disease (2), Alzheimer (3) and diabetes (4) and also the side effects of the long-term use of drugs on depressed patients (5), usage of therapeutic methods based on Complementary and Alternative Medicine (CAM) including Iranian Traditional Medicine (ITM) has been taken into consideration (5). ITM involves several non-pharmacological methods which one of the most notable of them is food therapy (6). Although the effectiveness of diets such as Mediterranean diet on preventing depression (7) or reducing its complications (8) have been studied in the literature, researches which specifically introduce effective foodstuff are very limited. Therefore, the aim of the present study was to introduce some food-based strategies, especially foodstuff for depression management based on ITM. This paper is organized in the following manner; sections 3.1 and 3.2 respectively are devoted to materials and methods of ITM and modern medicine. In section 4.1, the results obtained based on ITM and in section 4.2 the results obtained based on modern medicine are presented. Finally, the results are discussed and conclusions are made in section 5.

## 2. Objectives

Food-based strategies, which are one of the outstanding medical solutions in CAM including Iranian Traditional Medicine have been investigated.

## 3. Materials and Methods

### 3.1. Materials and Methods in ITM

Since in ITM resources, “depression” has not been determined as a distinct heading, the related materials were extracted in several steps. These steps can be categorized as follows:

Step one: seven keywords “Jaza”, “hozn”, “khobs-e-nafs” (in ITM this means sadness without cause), “Gham”, “Karb”, “Vasvas” (“Vasvas” in ITM is different from obsession and means suspicion) and “Melancholia” were selected. These keywords are related to depression based on their definitions.

Step two: all parts of important ITM sources namely Kamel al-Sanaat al-Tibbyyah, Al-Qanun fi al-Tibb and Zakhireh Kharazmshahi, were studied and the related issues were extracted by using the aforementioned keywords.

Step three: in extracting the related contents, we were faced with two different applications for each of the mentioned keywords. They were used in some cases “as a symptom” and in some other cases “as a cause”. For example, “melancholy” may lead to the state of “Gam”. In this case “Gam” is a symptom (9-11). On the other hand, “Gam” can lead to indigestion. In this case “Gam” is a cause (10). Therefore, those cases in which the mentioned keywords had been used “as a cause” and involved psychosomatic disorders were put aside. Next, food strategies were extracted based on cases in which the mentioned keywords had been used “as a symptom”.

Step four: in this step the selected food strategies in step three were classified into prevention and treatment parts. For example if the moods of sadness and immerse appear in someone without an external cause and this person does not receive any attention, such conditions may lead to melancholy (9). Therefore, food strategies for melancholy were situated in the treatment part. On the other hand, moods of sadness typically exist in a melancholic patient (9-11). Therefore, food strategies for melancholy were part of the treatment section.

Step five: after separation of the prevention and treatment parts, three categories were considered for the extracted food strategies: a) Food strategies related to titles in which the keywords were symptoms of a general physical disease whose origin was the brain, heart, liver, stomach, spleen or uterus. b) Food strategies related to titles in which the keywords were independent symptoms by themselves. For example, if someone is accustomed to have only one meal a day but unlike his habit he eats two meals a day, a state of sadness without a cause will catch him (10). Here, sadness does not happen because of a disease and it is considered as an independent symptom. c) Food strategies related to melancholy. In this step, in order to use the most specialized findings for this paper the main part of the extracted content was put aside and only parts b and c were selected.

Step six: food strategies, selected through the described method, were arranged under three headings, including; singular foodstuffs, combined foodstuffs, and nutritional rules.

Step seven: under each heading prohibited and prescribed cases were separated.

Step eight: finally for the ease of use, among the entire singular and combined foodstuffs only foodstuffs, which are usually available in today’s societies, were selected.

### 3.2. Materials and Methods in Modern Medicine

In this review study, databases including PubMed and Magiran were searched for related studies by using fourteen keywords including; depression, depressive, mood, antidepressant, antidepressive, nutrition, nutritional, diet, meal, food, functional food, healthy food, healthy diet, medicinal food and also scientific and English terms of all singular foodstuffs and some combined foodstuffs, which are introduced in this paper. In the search procedure, appropriate Booleans and truncations were also used to retrieve the best queries. In the first step, only human studies done during the time period between 2000 and 2012 were selected. In cases where human studies were not available, inevitably the search was repeated again for animal studies. Thus, 250 papers were retrieved in the primary search. In the next step, 38 papers were selected after screening the titles. It should be noted that the accompaniment of depression with other diseases was considered as an exclusion criterion.

Due to the extensive domain of the study, care must be taken to select exactly related articles as much as possible. To this end, the objective and main findings of each paper was carefully studied and relevance was assessed as “exactly related”, “related” and “less related”. Ultimately, 17 papers did not possess good relevance and only 21 papers were selected. The type of each study was considered as a criterion for quality assessment but due to the low number of papers ultimately selected, all types of studies including “randomized controlled trial” (RCT), “cohort”, “case-control” and “cross-sectional” other than “reports” were considered in this study. Consequently, according to our findings all of the selected articles in the relevance assessment were also chosen in the quality assessment. It should be noted that all of the papers were reviewed twice but by only one reviewer. Since some of the combined foodstuffs belonged merely to the Persian culture, it was impossible to search for findings related to such foodstuffs in databases. Also, it was impossible to search modern medicine databases for concepts such as “Sodazaee” (defined as causing black bile), “Safrazaee” (defined as causing yellow bile), “warmness”, “coldness”, “wetness”, and also being “Mofarrah” (defined as enlivening) because they are only defined in ITM.

## 4. Results

### 4.1. Results Based on ITM

In this section, food-based strategies for depression management presented in ITM are summarized as follows.

increasing amounts of food for a person who is accustomed to eat a few amounts of food (9), eating meal befor the accustomed time of each meal (9), consuming two meals a day for a person who is used to consume one meal a day (10), and consuming “Sodaza” foodstuffs (9).

consuming foods causing “warmness” and “wetness” such as “Esfidba” (9) which is cooked lamb with onion and chickpeas.

Consuming “Safraza” foodstuffs (9), Consuming “Sodaza” foodstuffs (9), consuming food before elimination of dyspepsia symptoms following the last meal (10), consuming salty foodstuffs (10), consuming tart foodstuffs (10), and consuming highly fried foods (11).

Treatment / prohibition / singular foodstuffs: onion (9), garlic (9), mustard (9), vinegar (9), cabbage (9, 10), beef (9), lentil (9, 10), and broad bean (10).

Treatment / prohibition / combined foodstuffs: foodstuffs modified with honey (9), bread with plenty of barn (9), and corned beef (9).

Treatment / prescription / nutrition rules: consuming foods causing “wetness” (9, 11), hypnotic foods (9), foods causing “warmness” and “wetness” (10), meats that can be digested quickly (10), suitable foods that cause fatness (10), fatty and sweet foodstuffs (10), moderate amounts of food chosen according to the person’s digestion ability (11), food and drinks with “cold” and “wet” nature (11), one type of food in each meal (11), and “Mofarrah” foods with a “warm” nature (11).

Treatment / prescription / singular foodstuffs: currant (9), spinach (9, 11), watermelon (9), sweet apple (9), dried fig (9), lettuce (9, 11), peach (9), pomegranate (9), rainbow trout (9, 10), slightly fried egg (9, 11), grape (9), pistachio (9), oregano (9), squash (9), cucumber marrow (9), young lamb (9), almond (9), bananas (9), mint (9), chard (11), celery (11), chicory (11), and milk (10).

Treatment / prescription / combined foodstuffs: officinal beer (9) which is a filtrated liquid produced from boiling 1 unit of barley (without bran) with 14 units of water, “Esfidba” with shoulder lamb (9), shoulder lamb gently fried with olive oil or almond oil (9), shoulder lamb cooked with squash, spinach or lettuce (9), “Esfidba” with chicken meat (9), “Zirba” with chicken meat (9) (“Zirba” is a kind of stew cooked with vinegar, saffron, cumin and materials with a sweet taste), white bread (9), chicken meat cooked with squash or spinach (9), and lamb’s leg and neck cooked with water and salt (11).

### 4.2. Results Based on Modern Medicine

In this section, related results for the mentioned food strategies have been arranged in two categories as follows:

Diets: the relationship between mood and nutrition has been shown by several studies. For example consuming fast foods and commercial baked goods increase the risk of depression (12, 13). Also, the results of existing studies indicate a direct connection between depression and diets containing processed or fried foods, refined grains, sugary products and beer (14). On the other hand, the positive effects of a Mediterranean diet, which includes vegetables, fruits, grains, frijol, nuts, dairy, olive oil and aquatic food has been shown to prevent depression. These foodstuffs are important sources of nutrients linked for depression prevention (7). Previous research (15) has indicated that the nutrition recommendations based on the Australian guidelines for healthy eating with particular attention to fruits, vegetables and whole grains have a good effect on depressed patients. Also, it has been indicated that in addition to the benefits of moderate-sodium dietary approaches that stop the negative effects of hypertension (DASH type diet) on blood presser and bone health, this diet also has positive effects on improving mood. This low fat diet includes lean red meat, wholegrain breads and cereal (16). A previous study (17), indicated that the dietary predictors of depression prevalence are similar to those that predict coronary heart disease and diabetes and as already mentioned such illnesses have a greater prevalence in depressed patients (2, 4).

Foodstuffs: many studies have been done regarding the effectiveness of fish, as a good source of omega-3 fatty acids, on depression (18). Two previous researches (19-21) showed that the low consumption of fish results in a depressed mood in men yet in another study the low frequency of fish consumption had an association with depression in women, but not in men (22). In an animal study (23), the mechanisms of the antidepressant-like effects of garlic were demonstrated. Furthermore, it has been shown (24, 25) that milk consumption effectively improves depressed mood. In a study conducted on animal models, oregano was introduced as a brain activator (26). The impacts of mint on increasing alertness and memory have been demonstrated (27). In an animal model, spinach caused increased levels of brain serotonin and decreased levels of norepinephrine and dopamine (28). The antidepressant effects of saffron (used in drug-based strategies for depression management) have also been shown by previous studies (29, 30).

## 5. Discussion

Among thirty cases of prescribed and prohibited singular foodstuffs in ITM manuscripts, only the impacts of six foodstuffs, including fish, garlic, milk, oregano, mint and spinach on depression have been studied in the literature of modern medicine (18-28). As previously cited (19-21), the low consumption of fish results in a depressed mood in men however in one study the low frequency of fish consumption was associated with depression in women but not men (22). The difference between the positive effects of fish consumption on depression in men and women has not been directly expressed in ITM resources. From the perspective of these resources, this deference could be considered as a result of different temperaments of these two sexes. In ITM texts consuming garlic has been prohibited (9) but in modern medicine databases its antidepressant-like effects have been reported (23). Investigating the reason for this conflict can be done in a separate study.

It is important to note that the consumption of onion (9), garlic (9), cabbage (9, 10), lentil (9, 10) and broad bean (10) has been forbidden in ITM resources while these foodstuffs are among vegetables and frijol groups which are prescribed by the Mediterranean diet without any exceptions (7). This contradiction can be regarded as a sense of applied precision in the manuscripts of ITM. The impact of highly fried food consumption on depression incidence has been indicated by recent findings (14). Similarly the consumption of such food has also been prohibited in ITM texts (11). The negative effects of processed pastries and sugary products on depression has been approved by modern studies (12, 14). On the other hand in ITM texts, the consumption of fatty and sweet foods has been recommended for depression management (10) but the consumption of foodstuff modified with honey has been forbidden in these texts (9). Thus, it may be suggested that in ITM texts, the recommendation for consuming fatty and sweet foods only includes natural foods with little sweetness.

The negative effect of non-bran grains consumption on depression has been approved by recent investigations (14) while consuming non-bran bread has been recommended by ITM resources (9). Regarding the reason for this incompatibility it can be said that in the past the problem of constipation was not as prevalent as today, while constipation is one of the most important problems in today’s societies (31). Accordingly, the prescription of whole bread, which has more fiber than non-bran bread, is helpful for resolving constipation and thereby it can be effective in reducing depression symptoms. Another reason for the mentioned conflict could be the greater content of light bread regarding other nutrients such as omega-3 fatty acids and tryptophan (32) that effect depression management. Therefore consumption of light bread increases the intake of these nutrients compared to whole bread. Perhaps the prescription of light bread in ITM manuscripts can be justified through these perspectives. As previously mentioned ITM texts recommend the consumption of fatty and sweet foods for depression management (10). On the other hand, a previous study (16), showed that DASH type diet as a low fat diet has a positive effect on improving mood. This paradox may possibly be resolved considering the type of fat intended in these two diet strategies. Also, some other probabilities such as the huge difference between new age and olden day lifestyles should not be ignored.

Some issues such as the prohibition of increasing amounts of food for a person who is accustomed to eat a few amounts of food (9), prohibition of eating food before the accustomed time of each meal (9), prohibition of eating two meals in a day for a person who is accustomed to eat only one meal a day (10), prohibition of having a meal before elimination of dyspepsia symptoms following the last meal (10), prohibition of eating tart foods (10), prescription of eating hypnotic foods (9), eating appropriate foods which are fattening (10), eating moderate amounts of food chosen according to the person’s digestion ability (11), and eating only one kind of food in each meal (11) were presented in ITM texts. However, due to the importance and extension of the mentioned issues, it is necessary to investigate these cases in separate studies. It should be noted that because of some restrictions such as undefined concepts of humors in modern medicine and some cultural features in relation to the combined foods it was impossible to verify a significant part of the presented strategies in association with recent findings. Also, the existing findings for the singular foodstuffs and diets were very limited. An appropriate approach to eliminate these constraints would be designing different clinical studies for such these cases. Considering the mentioned points, complementary studies can be conducted in relation to this subject and consequently more documented health-promoting foods (33) and behaviors can be introduced for patients suffering from depression.
